# Non-equilibrium estimates of gene flow inferred from nuclear genealogies suggest that Iberian and North African wall lizards (*Podarcis *spp.) are an assemblage of incipient species

**DOI:** 10.1186/1471-2148-8-63

**Published:** 2008-02-26

**Authors:** Catarina Pinho, D James Harris, Nuno Ferrand

**Affiliations:** 1 CIBIO, Centro de Investigação em Biodiversidade e Recursos Genéticos. Campus Agrário de Vairão, 4485-661 Vairão, Portugal; 2 Department of Genetics, Rutgers, the State University of New Jersey. Piscataway, NJ 08854, USA; 3 Departmento de Zoologia e Antropologia, Faculdade de Ciências da Universidade do Porto, Praça Gomes Teixeira, 4099-002 Porto, Portugal

## Abstract

**Background:**

The study of recently-diverged species offers significant challenges both in the definition of evolutionary entities and in the estimation of gene flow among them. Iberian and North African wall lizards (*Podarcis*) constitute a cryptic species complex for which previous assessments of mitochondrial DNA (mtDNA) and allozyme variation are concordant in describing the existence of several highly differentiated evolutionary units. However, these studies report important differences suggesting the occurrence of gene flow among forms. Here we study sequence variation in two nuclear introns, *β-fibint7 and 6-Pgdint7*, to further investigate overall evolutionary dynamics and test hypotheses related to species delimitation within this complex.

**Results:**

Both nuclear gene genealogies fail to define species as monophyletic. To discriminate between the effects of incomplete lineage sorting and gene flow in setting this pattern, we estimated migration rates among species using both *F*_*ST*_-based estimators of gene flow, which assume migration-drift equilibrium, and a coalescent approach based on a model of divergence with gene flow. Equilibrium estimates of gene flow suggest widespread introgression between species, but coalescent estimates describe virtually zero admixture between most (but not all) species pairs. This suggests that although gene flow among forms may have occurred the main cause for species polyphyly is incomplete lineage sorting, implying that most forms have been isolated since their divergence. This observation is therefore in accordance with previous reports of strong differentiation based on mtDNA and allozyme data.

**Conclusion:**

These results corroborate most forms of Iberian and North African *Podarcis *as differentiated, although incipient, species, supporting a gradual view of speciation, according to which species may persist as distinct despite some permeability to genetic exchange and without having clearly definable genetic boundaries. Additionally, this study constitutes a warning against the misuse of equilibrium estimates of migration among recently-diverged groups.

## Background

The study of emerging species poses several challenges for evolutionary biologists. From a phylogenetic perspective, for example, attempts to reconstruct relationships among such taxa are often hampered by a poor resolution of relationships, by lack of monophyly inferred from individual gene genealogies due to incomplete lineage sorting or, when multiple loci are analysed, by discordant scenarios portrayed by distinct genealogies. When a species splits into two, the diverging forms will share much of their genetic variation for a long period of time and the pre-existing polymorphism may be sorted in a stochastic fashion or reflect differential selective pressures, not necessarily tracking species-splitting events. Moreover, closely-related species are likely to retain some permeability to the exchange of genes, which may affect some loci more than others [1,2]. These features become even more striking when more than two species are involved; in rapidly radiating taxa the chance of incomplete lineage sorting increases and complex patterns of admixture often arise, complicating the recognition of species boundaries in the context of both biological and phylogenetic species definition criteria [3-6]. As a consequence, an additional complication arises when attempting to estimate levels of gene flow among such nascent species because of the need to discriminate between the amount of polymorphism that is shared due to incomplete lineage sorting and to actual genetic exchange. In fact, several widely-used estimates of gene flow such as Wright's [7] equation relating *F*_*ST *_and the product *Nm *assume equilibrium migration and therefore may not perform well in species that are still undergoing lineage sorting and therefore violate this assumption [8-10]. Recent analytical methods [10,11], in contrast, do not assume equilibrium and allow the analysis of gene flow and divergence in the same framework, thus being appropriate tools to evaluate migration rates among recently diverged taxa.

*Podarcis *wall lizards in the Iberian Peninsula and North Africa constitute a cryptic species complex that has been studied using both mtDNA [12-15] and allozyme data [16-18]. In concordance with emerging morphological evidence [19-21] both types of markers have documented the existence of several highly differentiated population groups, some of which correspond to the currently accepted species (*P. bocagei, P. carbonelli *and *P. vaucheri*), whereas others constitute different forms within the polytypic and, from a mitochondrial perspective, paraphyletic *P. hispanica*. Reported genetic distances at the mitochondrial level are higher than traditional species-delimitation thresholds proposed for squamates [22]. However, not all the mitochondrial DNA lineages seem to belong to clearly distinct morphological entities [23]. Moreover, although both nuclear and mitochondrial markers provide roughly concordant species delimitation, a detailed comparison of both analyses reports two significant differences [18]. First, not all of the forms detected using mtDNA are readily distinguishable using allozymes, which could result either from massive introgression of the nuclear genome or from the low number of loci studied coupled with low overall diversity levels observed in allozymes, limiting their ability to detect subtle population structure. Secondly, evolutionary relationships between species and forms within *P. hispanica *as depicted by mtDNA are very well supported, suggesting a "step-by-step" speciation scenario, whereas the analyses of allozymes produces only a few well-supported multi-species clusters, implying a radiation scenario. These discordant results were interpreted as reflecting the shorter time required by mtDNA to achieve monophyly [18]. Despite their utility in this case of corroborating the major partitions inferred from mtDNA, allozyme analyses have several drawbacks, of which the most important for comparing the patterns of mitochondrial and nuclear levels of divergence is the lack of a genealogical framework to understand the relationships between alleles and the evolutionary processes underlying the distribution of genetic variation.

In this work we aim to analyse the evolutionary history of the Iberian and North African *Podarcis *species complex from a yet unexplored perspective: nuclear genealogies. We studied nucleotide variation at two nuclear introns in individuals representing all known morphotypes and mitochondrial lineages. Our main goals were: a) to investigate whether species and forms of *Podarcis *are monophyletic with respect to nuclear genealogies; b) if not, to assess the relative roles of incomplete lineage sorting and gene flow in shaping the observed patterns; and ultimately c) to understand if the genetic boundaries delimiting forms within the *Podarcis *species complex are well-defined. Our results evidence a striking contrast between monophyly of mitochondrial DNA and extreme polyphyly of nuclear genealogies. Moreover, because equilibrium-based, classic estimates of gene flow among forms are remarkably different from those estimated in a divergence, non-equilibrium framework, this study highlights the confounding effect that incomplete lineage sorting may have in migration rate estimates and consequent inference of species boundaries when recent divergence is not taken into account.

## Results

### Mitochondrial DNA assignment

**Figure 1:**
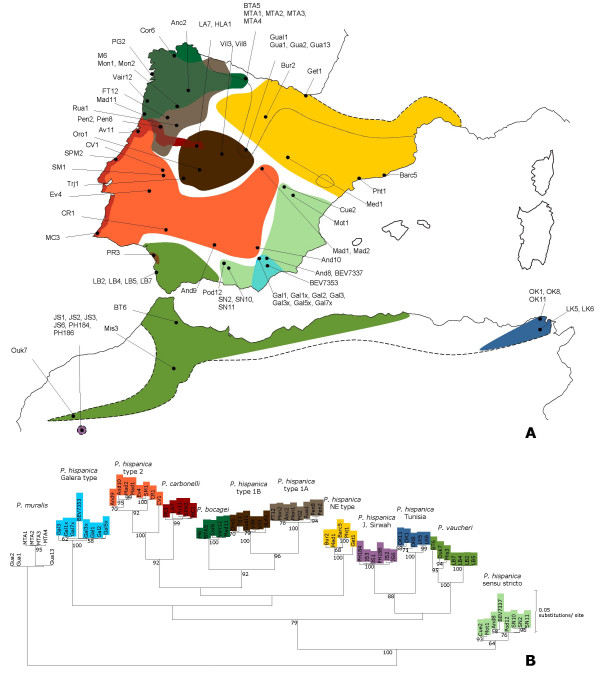
**Geographical origin and mitochondrial DNA assignment of samples used in this study**. **A**. Map of the Iberian Peninsula and the Maghreb showing the putative distribution of mtDNA lineages based on a compilation of available data and samples analysed in this study. The dashed line represents the distribution of the Iberian-Maghrebian clade of *Podarcis*; the dotted line represents the limits of the Iberian distribution of *P. muralis*. **B**. Maximum-likelihood tree of the mitochondrial gene ND4 for the samples analysed. Bootstrap values over 50% are shown.

In order to assign individuals to a mitochondrial DNA lineage, we sequenced a portion of the ND4 gene in all analysed individuals. The mitochondrial data set included 78 sequences and was trimmed to a common fragment of 534 bp. Previously unpublished sequences have been submitted to GenBank, accession numbers EU269551–EU269594. This data set contained 187 polymorphic positions, of which 179 were parsimony informative. None of these polymorphisms correspond to insertions or deletions, which conforms to the coding nature of the region analysed. An ML tree depicting the relationships between the observed haplotypes is shown in Figure 1. Because it includes less informative characters, the depicted tree does not reflect the same well-supported relationships that were inferred in a previous phylogenetic study including 2425 bp of mitochondrial DNA sequence data [15] although exactly the same major phylogroups were recovered. This tree was used to assign individuals to a mitochondrial group. All the individuals that had a prior morphological assignment clustered within the expected mitochondrial lineage, with the exception of the individual BEV7337, which morphologically resembles a "Galera type" individual (P.A. Crochet, pers. comm.) but that nevertheless clustered within the sympatric *P. hispanica *sensu stricto.

### Nuclear gene variability and recombination

We studied DNA sequence variation in two nuclear introns, *6-Pgdint7 *and *β-fibint7*, in individuals representing all known mtDNA lineages. Alignment of the *6-Pgdint7 *sequences for 136 chromosomes was trimmed to a common fragment of 503 bp. This 503 bp alignment required 10 insertions or deletions, from 1 bp to 23 bp long, and included a small poly-G repetitive region with size variability. Indels were inserted to maximise base pair identity in conserved sequenced blocks flanking the indel. A total of 65 different alleles were detected. Alignment of *β-fibint7 *was pruned to a common fragment of total length 951 bp for 140 chromosomes; this alignment required several insertions and deletions, including a very large (~350 bp) insertion in all samples bearing the "Galera" mtDNA lineage plus sample BEV7337 which was probably responsible for the failure to amplify the complete intron in these samples using primers BF8 and BFXF. At least 80 alleles were detected. The "Galera" insertion showed a poly-C/poly-T motif within it, with size variability, but each allele's number of repeats could not be resolved. Complete allele sequences have been submitted to GenBank, accession numbers EU269595–EU269659 (*6-Pgdint7*) and EU269471–EU269550 (*β-fibint7*). Correspondence between alleles, the samples in which they were detected and accession numbers is given in Additional File 1.

Polymorphism levels calculated from the data sets without alignment gaps are presented in Table 1. The nuclear loci analysed in this study, despite being introns, show polymorphism levels considerably lower than those observed in the mitochondrial DNA fragment analysed (e.g. π_6-*Pgdint*7 _= 0.01721; π_*β-fibint7 *_= 0.01269; π_*ND*4 _= 0.10738). Interestingly, both nuclear genes show significantly negative values of Tajima's D, indicating a skew towards rare alleles. Both genes failed to pass the four-gamete test of Hudson and Kaplan [24] and were inferred to have suffered several recombination events (at least 7 in the case of *6-Pgdint7 *and at least 8 in *β-fibint7*). However, when applying the more sensitive test of Bruen *et al. *[25] we were not able to reject the null hypothesis of no recombination (*P*-values for the permutation test shown in Table 1).

**Table 1:** Summary statistics and tests of recombination for the three gene regions analysed in this study. Alternative values of Tajima's D and *F*_ST _are for estimates including or excluding *P. muralis*. N, number of haplotypes sequenced; *H*, number of haplotypes detected. S, number of segregating sites; π, nucleotide diversity; θ, population mutation parameter, calculated according to Watterson [64]; Rm, minimum number of recombination events [24]; Φ_*w *_statistic p-value calculated according to Bruen et al. [25].

Gene	Taxa	Length (bp)	N	Polymorphism				Tajima's D	Recombination		***F***_ST_
							
				*H*	*S*	*π*	θ		Rm	Φ_*w *_*P*-value	
ND4	All	534	78	54	187	0.10738	0.07107	0.67941/0.65100	-	-	0.89885/0.89707
	*P. bocagei*		5	3	2	0.00187	0.00180	0.24314			
	*P. carbonelli*		5	5	7	0.00562	0.00629	-0.74682			
	*P. vaucheri*		7	4	32	0.02836	0.02446	0.45857			
	*P. hispanica *type 1A		8	3	5	0.00461	0.00361	1.26023			
	*P. hispanica *type 1B		6	5	9	0.00687	0.00738	-0.41545			
	*P. hispanica *type 2		8	7	45	0.03023	0.03250	-0.48372			
	*P. hispanica *sensu stricto		8	6	17	0.01318	0.01228	0.37704			
	*P. hispanica *type 3		5	4	14	0.01273	0.01258	-0.40617			
	*P. hispanica *Galera		8	5	18	0.00876	0.01300	-1.68765*			
	*P. hispanica *Jebel Sirwah		6	4	3	0.00225	0.00246	-0.44736			
	*P. hispanica *Tunísia		5	4	9	0.00974	0.00809	1.44761			
	*P. muralis*		7	4	14	0.01427	0.01070	1.86716			
*6-Pgdint7*	All	414	136	60	91	0.01721	0.04007	-1.89302**/-2.02372*	7	0.276	0.61498/0.46435
	*P. bocagei*		10	2	1	0.00129	0.00085	1.30268			
	*P. carbonelli*		10	5	7	0.00644	0.00598	0.46601			
	*P. vaucheri*		12	7	11	0.00659	0.00880	-0.87808			
	*P. hispanica *type 1A		12	6	11	0.00853	0.00880	-0.04145			
	*P. hispanica *type 1B		10	8	16	0.01390	0.01366	-0.03458			
	*P. hispanica *type 2		14	10	24	0.01680	0.01823	-0.62704			
	*P. hispanica *sensu stricto		14	9	15	0.00805	0.00993	-0.77168			
	*P. hispanica *type 3		10	7	12	0.00859	0.01025	-0.49424			
	*P. hispanica *Galera		12	3	3	0.00131	0.00205	-1.17901			
	*P. hispanica *Jebel Sirwah		12	3	6	0.00677	0.00480	1.63651			
	*P. hispanica *Tunísia		10	6	4	0.00462	0.00512	-0.40924			
	*P. muralis*		10	3	2	0.00215	0.00171	0.83017			
*β-fibint7*	All	507	140	67	100	0.01269	0.03576	-2.15863**/-2.29815**	8	0.194	0.51125/0.40491
	*P. bocagei*		10	4	5	0.00197	0.00349	-1.74110*			
	*P. carbonelli*		10	7	18	0.01267	0.01255	-0.20511			
	*P. vaucheri*		10	6	20	0.01179	0.01394	-0.72606			
	*P. hispanica *type 1A		16	11	25	0.00830	0.01486	-1.72813			
	*P. hispanica *type 1B		12	8	13	0.00529	0.08490	-1.59698			
	*P. hispanica *type 2		12^+^	8	18	0.01133	0.01176	-0.15948			
	*P. hispanica *sensu stricto		14	9	16	0.00759	0.01013	-0.61276			
	*P. hispanica *type 3		10	7	20	0.01315	0.01394	-0.26800			
	*P. hispanica *Galera		12	6	9	0.00493	0.00588	-1.30703			
	*P. hispanica *Jebel Sirwah		12	2	1	0.00033	0.00065	-1.14053			
	*P. hispanica *Tunísia		10	1	0	0.00000	0.00000	-			
	*P. muralis*		12	2	1	0.00033	0.00065	-1.14053			

* *p *< 0.05; ** *p *< 0.01; ^+^2 alleles were discarded because of a very large deletion.

### Nuclear gene genealogies

**Figure 2:**
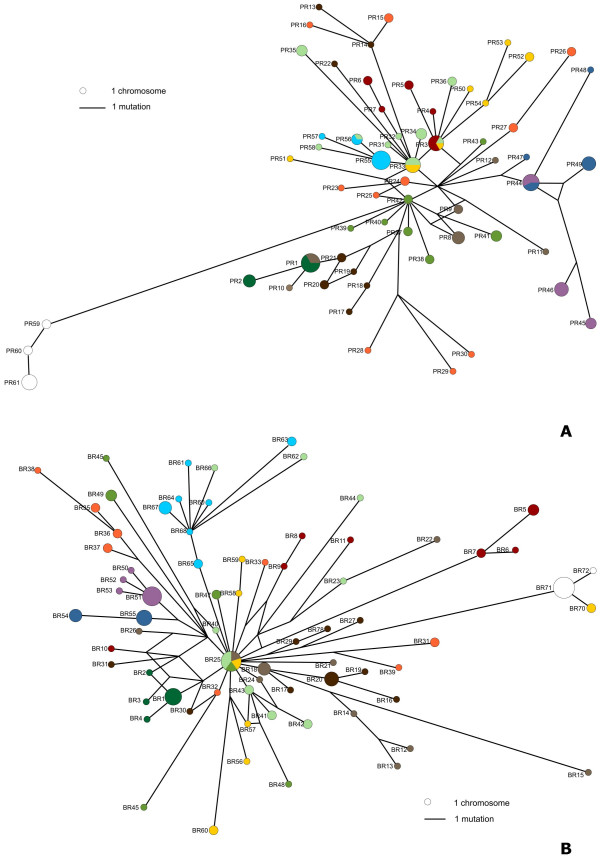
**Gene genealogies for two nuclear introns in Iberian and North African *Podarcis***. Allele names correspond to those in table 5. Colours represent mitochondrial DNA lineages and correspond to those used in figure 1. Alleles detected in *P. muralis *are represented in white. **A**. *6-Pgdint7*. **B**. *β-fibint7*.

The haplotype networks inferred for both nuclear genes are shown in Figure 2. These networks were built after removing all but the first base of each indel (see Methods), which resulted in 100 variable characters and 61 alleles in *6-Pgdint7 *and 113 characters and 72 alleles in *β-fibint7*. The correspondence between alleles represented in the haplotype networks and the complete alleles that have been deposited in GenBank is given in Additional File 1.

The most evident result is a complete lack of monophyly of the mitochondrial-defined groups. Many alleles are even shared by distinct species. Moreover, although the outgroup *P. muralis *represents a relatively distinct lineage according to both genes, the group formed by all Iberian and North African *Podarcis *is not monophyletic with respect to this species according to the *β-fibint7 *genealogy, since one individual carrying *P. hispanica *type 3 mtDNA exhibits an allele that falls within the clade formed by *P. muralis*.

Because this lack of monophyly could result from an insufficiency of data to recover correct relationships, we tested alternative hypotheses based on mitochondrial DNA phylogenetic relationships using a Shimodaira-Hasegawa test [26]. Using datasets without removing indels (but excluding the poly-C/poly-T region in "Galera" samples), we enforced two distinct topological constraints for each gene: i) monophyly of all species; ii) monophyly of the three major groups recovered for Iberian and North African *Podarcis *by Pinho *et al. *[15] (group 1: *P. bocagei, P. carbonelli*, *P. hispanica *type 1A and 1B, *P. hispanica *type 2; group 2: *P. hispanica *sensu stricto, *P. vaucheri*, *P. hispanica *Tunisia and *P. hispanica *Jebel Sirwah; group 3: *P. hispanica *type 3 and *P. hispanica *Galera). In both of these analyses *P. muralis *was also included and constrained to monophyly. Because a few cases of allele proximity could at a first glance be interpreted as suggestions of recent gene flow, we also conducted the same analyses excluding these alleles (BEV7337A and BEV7337B in *6-Pgdint7*; BEV7337A, BEV7337B, Get1A and Get1B in *β-fibint7*). All these tests suggest that enforced topologies are significantly less likely than the ones observed (*p *< 0.01), with the exception of the division into three groups in *β-fibint7 *excluding the putative introgressed alleles.

### Genetic differentiation and equilibrium gene flow estimates between groups

We evaluated overall genetic differentiation among mtDNA-defined species by computing *F*_ST _for the two data sets. These results are shown in Table 1. Values obtained for the mitochondrial DNA are also shown for comparison (noting, however, that "species" are, by definition, monophyletic with respect to the mtDNA, leading to necessarily high levels of differentiation). Despite the lack of monophyly, differentiation between the 12 species accounts for around 61% of genetic variation in *6-Pgdint7 *and 51% in *β-fibint7*; these values fall to around 46% and 40%, respectively, when the outgroup *P. muralis *is excluded from the analyses.

Examination of pairwise *Nm *values in more detail (Table 2) reveals distinct trends across species pairs; in general *Nm *values based on nuclear genes are different from 0, ranging from 0.01 (between *P. muralis *and both *P. bocagei *and *P. hispanica *Tunisian type) to 1.76 (between *P. hispanica *types 1A and 1B). Besides this last case, other species pairs exhibit *Nm *values higher than 1, suggesting large levels of gene flow under this model: *P. vaucheri *vs. *P. hispanica *types 1A and 1B, *P. hispanica *type 1B vs. *P. hispanica *type 2 and *P. hispanica *sensu stricto vs. *P. hispanica *type 3.

**Table 2:** Pairwise *F*_ST _and corresponding *Nm *values [7] between species of Iberian and North African *Podarcis*. *F*_ST _and *Nm *values are shown below and above the diagonal, respectively. *Nm v*alues higher than 1 are shown in bold. Species names are abbreviated as follows:*Pb *– *P. bocagei*; *Pc *– *P. carbonelli*; *Pv *– *P. vaucheri*; *Ph*1A – *P. hispanica *type 1A; *Ph*1B – *P. hispanica *type 1B; *Ph*2 – *P. hispanica *type 2; *Ph*ss – *P. hispanica *sensu stricto; *Ph*3 – *P. hispanica *type 3; *Ph*Gal – *P. hispanica *Galera type; *Ph*JS – *P. hispanica *Jebel Sirwah type; *Ph*Tun – *P. hispanica *Tunisian type; *Pm *– *P. muralis*.

		*Pb*	*Pc*	*Pv*	***Ph*1A**	***Ph*1B**	***Ph*2**	***Ph*ss**	***Ph*3**	***Ph*Gal**	***Ph*JS**	***Ph*Tun**	*Pm*
***Pb***	mtDNA	-	0.02	0.09	0.02	0.03	0.11	0.04	0.04	0.03	0.01	0.03	0.04
	nDNA	-	0.15	0.28	0.51	0.43	0.32	0.22	0.24	0.07	0.06	0.04	0.01
***Pc***	mtDNA	0.9649	-	0.10	0.02	0.03	0.14	0.06	0.05	0.03	0.02	0.03	0.04
	nDNA	0.6240	-	0.36	0.38	0.39	0.48	0.73	0.87	0.22	0.18	0.15	0.05
***Pv***	mtDNA	0.8428	0.8277	-	0.08	0.09	0.18	0.14	0.13	0.09	0.11	0.12	0.09
	nDNA	0.4728	0.4084	-	**1.69**	**1.19**	0.89	0.66	0.69	0.23	0.26	0.25	0.06
***Ph*1A**	mtDNA	0.9572	0.9565	0.8649	-	0.05	0.09	0.04	0.04	0.03	0.02	0.02	0.04
	nDNA	0.3274	0.3952	0.1288	-	**1.76**	0.79	0.79	0.73	0.25	0.25	0.25	0.07
***Ph*1B**	mtDNA	0.9348	0.9432	0.8435	0.9072	-	0.10	0.05	0.05	0.03	0.02	0.03	0.04
	nDNA	0.3674	0.3880	0.1742	0.1244	-	**1.02**	0.88	0.89	0.25	0.24	0.23	0.06
***Ph*2**	mtDNA	0.8175	0.7790	0.7380	0.8522	0.8265	-	0.13	0.12	0.08	0.09	0.10	0.10
	nDNA	0.4391	0.3427	0.2187	0.2413	0.1965	-	0.69	0.72	0.32	0.33	0.33	0.08
***Ph*ss**	mtDNA	0.9186	0.8919	0.7858	0.9253	0.9028	0.7986	-	0.07	0.05	0.05	0.06	0.07
	nDNA	0.5286	0.2550	0.2752	0.2404	0.2206	0.2651	-	**1.64**	0.51	0.20	0.20	0.05
***Ph*3**	mtDNA	0.9235	0.9089	0.7966	0.9282	0.9079	0.8006	0.8760	-	0.06	0.03	0.06	0.05
	nDNA	0.5119	0.2236	0.2648	0.2557	0.2200	0.2568	0.1322	-	0.34	0.22	0.22	0.08
***Ph*Gal**	mtDNA	0.9389	0.9373	0.8454	0.9426	0.9360	0.8578	0.9090	0.8982	-	0.03	0.04	0.04
	nDNA	0.7910	0.5367	0.5255	0.5041	0.5038	0.4352	0.3299	0.4240	-	0.08	0.07	0.02
***Ph*JS**	mtDNA	0.9787	0.9685	0.8143	0.9703	0.9614	0.8496	0.9113	0.9349	0.9523	-	0.04	0.04
	nDNA	0.8123	0.5837	0.4924	0.4974	0.5132	0.4324	0.5534	0.5272	0.7614	-	0.12	0.02
***Ph*Tun**	mtDNA	0.9471	0.9453	0.8044	0.9529	0.9368	0.8283	0.8867	0.8963	0.9322	0.9276	-	0.05
	nDNA	0.8492	0.6325	0.4977	0.5034	0.5229	0.4287	0.5603	0.5328	0.7892	0.6794	-	0.01
***Pm***	mtDNA	0.9321	0.9237	0.8414	0.9310	0.9189	0.8387	0.8740	0.9025	0.9229	0.9338	0.9169	-
	nDNA	0.9498	0.8372	0.8094	0.7928	0.8164	0.7576	0.8265	0.7494	0.9198	0.9381	0.9557	-

### Application of the isolation with migration model to Iberian and North African *Podarcis*

Independent runs using IM converged on approximate marginal posterior probability distributions. We were able to obtain reliable estimates for θ1, θ2 and migration rates across most of the tested pairs; however, some 90% highest posterior density (HPD) intervals could not be reliably assessed either for θ or migration. The posterior probability distributions for the ancestral population sizes were generally flat or had a maximum at the lowest bin; our data do not therefore seem to incorporate enough information for these estimates to be reliable. Time since divergence scaled by the mutation rate (t) was also not recovered with confidence for a few species pairs because the distributions were flat (albeit non-zero), particularly in the comparisons involving species that are reciprocally monophyletic for both genes. Most of the curves, however, were well resolved or presented a clear peak although their tails did not reach 0; in these cases we report a value for the HPD, without obtaining a reliable 90% HPD interval.

In order to convert IM estimates into biologically meaningful values (effective population sizes, divergence times in years or migration rates) one needs an estimate of mutation rate. For the two genes included in the analyses, we have no information regarding the mutation rate and no straightforward way of calibrating a molecular clock. We therefore focused on estimates that do not require the assumption of a particular evolutionary rate: the population migration rate (*2Nm*) and estimates of relative divergence times (t = *tμ*) between species pairs. These results are given in Tables 3 and 4, respectively. In these tables, results involving *P. hispanica *sensu stricto and *P. hispanica *type 3 were estimated excluding alleles presumably introgressed from *P. hispanica *Galera type and *P. muralis*, respectively (obviously, the comparisons with the species from which the alleles were introgressed were performed on the complete data sets). Confirmation that these alleles and not others were introgressed from the other species was obtained when runs excluding them yielded migration rates of 0, in contrast to the levels estimated when they were present. In other cases of introgression, alleles that were obviously introgressed could not be a priori pinpointed with confidence.

**Table 3:** ML estimates and 90% HPD of population migration rates (*2Nm*) between species of wall lizards. *θ *and *m *parameters used to calculate migration rates were estimated using the IM software. Values higher than 0.1 are highlighted. Species abbreviations are as in table 2.

		from											
		*Pb*	*Pc*	*Pv*	***Ph*1A**	***Ph*1B**	***Ph*2**	***Ph*ss**	***Ph*3**	***Ph*Gal**	***Ph*JS**	***Ph*Tun**	*Pm*
**to**	*Pb*	-	0.001	0.001	0.002	0.001	0.001	0.001	0.001	0.001	0.004	0.001	0.001
			0.000 – 1.232^+^	0.000 – 0.800	0.000 – 2.868^+^	0.000 – 1.753^+^	0.000 – 0.887	0.000 – 0.928	0.000 – 1.110	0.000 – 0.646	0.000 – 0.752	0.000 – 0.782	0.000 – 0.354
	*Pc*	0.037	-	0.005	0.066	**0.127**	0.006	0.004	0.005	0.005	0.006	0.006	0.006
		0.002 – 2.154		0.002 – 1.593	0.002 – 1.870	0.002 – 1.916	0.002 – 1.503	0.002 – 5.057^+^	0.001 – 7.860^+^	0.003 – 1.883	0.003 – 1.893	0.003 – 1.983	0.003 – 0.617
	*Pv*	0.016	0.012	-	0.015	0.008	0.011	1.218*	**0.402**	0.010	0.009	0.009	0.009
		0.006 – 12.359	0.005 – 3.413		0.006 – 12.032	0.001 – 18.631^+^	0.004 – 2.354	0.003 – 9.669	0.004 – 52.568^+^‡	0.004 – 8.074‡	0.004 – 2.748	0.004 – 3.761	0.005 – 1.119
	*Ph*1A	7.501*	0.015	0.014	-	0.019	0.018	0.013	0.017	0.013	0.017	0.018	0.011
		0.009 – 51.669‡^+^	0.007 – 2.520	0.002 – 28.035^+^		0.006 – 28.481‡	0.010 – 1.942	0.006 – 10.953	0.009 – 5.504	0.007 – 2.034	0.008 – 16.823‡	0.008 – 15.027	0.006 – 0.853
	*Ph*1B	0.012	0.010	0.036	0.009	-	0.012	**0.338**	0.012	0.009	0.012	0.011	0.009
		0.006 – 25.531‡^+^	0.006 – 2.069	0.004 – 57.672‡^+^	0.003 – 7.664		0.006 – 3.894	0.004 – 20.194	0.005 – 7.917	0.005 – 2.436	0.005 – 5.222	0.005 – 5.445	0.005 – 0.876
	*Ph*2	0.018	0.019	0.018	0.017	0.016	-	0.017	0.017	0.018	0.018	0.017	0.015
		0.010 – 2.931	0.011 – 1.835	0.010 – 4.859	0.010 – 1.795	0.008 – 5.134		0.009 – 3.345	0.009 – 2.222	0.010 – 2.154	0.009 – 2.196	0.009 – 2.376	0.009 – 0.958
	*Ph*ss	0.005	0.054	0.005	0.004	0.005	0.005	-	0.003	1.212*	0.006	0.005	0.005
		0.002 – 2.065	0.002 – 5.791	0.002 – 2.362	0.002 – 1.448	0.002 – 6.000^+^	0.002 – 0.814		0.001 – 10.353^+^	0.129 – 6.020	0.003 – 2.067	0.002 – 1.719	0.003 – 0.501
	*Ph*3	0.012	0.012	0.007	0.008	0.011	0.010	0.015	-	0.013	0.009	0.009	**0.157**
		0.005 – 13.516‡	0.005 – 20.759‡	0.001 – 13.320	0.003 – 2.515	0.004 – 9.743	0.005 – 1.568	0.005 – 69.944‡^+^		0.005 – 10.353‡	0.004 – 6.933‡	0.004 – 9.892	0.004 – 2.130
	*Ph*Gal	0.002	0.002	0.002	0.002	0.002	0.000	0.002	0.002	-	0.002	0.002	0.002
		0.001 – 0.665	0.001 – 0.722	0.001 – 0.819	0.001 – 0.653	0.001 – 0.544	0.000 – 0.119	0.001 – 0.640	0.001 – 0.640		0.001 – 0.708	0.001 – 0.740	0.001 – 0.391
	*Ph*JS	0.001	0.001	0.001	0.000	0.001	0.001	0.001	0.001	0.001	-	**0.105**	0.001
		0.000 – 0.733	0.000 – 1.507	0.000 – 1.167	0.000 – 2.321^+^	0.000 – 1.431	0.000 – 0.727	0.000 – 0.899	0.000 – 1.171^+^	0.000 – 0.732		0.000 – 1.585^+^	0.000 – 0.277
	*Ph*Tun	0.003	0.001	0.001	0.001	0.001	0.001	0.001	0.001	0.001	0.002	-	0.001
		0.000 – 0.721	0.000 – 1.126	0.000 – 0.914	0.000 – 1.762^+^	0.000 – 0.864	0.000 – 0.712	0.000 – 0.900	0.000 – 0.995	0.000 – 0.752	0.000 – 1.149		0.000 – 0.305
	*Pm*	0.003	0.012	0.013	0.013	0.010	0.001	0.016	0.002	0.007	0.003	0.000	-
		0.000 – 0.267	0.000 – 0.347	0.000 – 0.524	0.000 – 0.468	0.000 – 0.454	0.000 – 0.445	0.000 – 0.354	0.000 – 0.676	0.000 – 0.264	0.000 – 0.219	0.000 – 0.228	

*: migration rate significantly different from 0 (*P *< 0.05).

‡, ^+^: the actual interval could not be reliably estimated because the likelihood surfaces for *θ *(‡) or *m *(^+^) were relatively flat.

**Table 4:** ML estimates and 90% HPD of time since divergence (t =*t*μ) between *Podarcis *species. Only estimates corresponding to a clear peak in the likelihood surface are shown. Species abbreviations are as in table 2.

	*Pb*	*Pc*	*Pv*	***Ph*1A**	***Ph*1B**	***Ph*2**	***Ph*ss**	***Ph*3**	***Ph*Gal**	***Ph*JS**	***Ph*Tun**	*Pm*
*Pb*	-											
*Pc*	?	-										
*Pv*	2.163	2.538	-									
	1.063 – 24.988*	1.063 – 7.863*										
*Ph*1A	?	3.163	2.363	-								
		1.363 – 5.613	1.588 – 3.163									
*Ph*1B	?	2.913	2.363	2.388	-							
		0.838 – 17.913*	0.363 – 8.238*	0.288 – 5.688*								
*Ph*2	3.313	3.838	3.388	3.438	2.788	-						
	0.738 – 15.288*	2.388 – 5.388	2.213 – 4.663	2.513 – 4.438	1.313 – 4.288							
*Ph*ss	1.963	1.763	2.163	2.438	2.188	2.888	-					
	1.013 – 24.788*	0.763 – 23.413*	1.013 – 23.138*	0.888 – 20.063*	0.683 – 21.688*	1.038 – 8.713*						
*Ph*3	1.988	1.788	2.063	2.663	2.313	2.938	1.638	-				
	0.738 – 23.163*	0.138 – 7.013*	0.688 – 10.263	1.538 – 3.963	1.488 – 3.363	1.788 – 4.263	0.588 – 23.113*					
*Ph*Gal	?	2.863	2.288	2.838	2.413	3.388	1.988	1.913	-			
		1.688 – 24.988*	1.938 – 14.638*	0.938 – 14.638*	0.813 – 22.038*	1.038 – 6.988*	1.133 – 24.988*	0.713 – 23.513*				
*Ph*JS	?	?	2.738	2.963	2.538	2.913	2.063	2.288	3.413	-		
			1.238 – 24.538*	1.238 – 24.988*	0.738 – 23.638*	0.713 – 10.913*	1.338 – 24.938*	0.838 – 22.463*	2.388 – 24.838*			
*Ph*Tun	?	?	2.363	2.738	2.488	3.038	2.038	2.113	3.338	?	-	
			1.063 – 23.538*	1.188 – 23.988*	0.688 – 22.863*	0.613 – 13.788*	1.163 – 24.588*	0.788 – 23.413*	2.063 – 24.937*			
*Pm*	?	?	?	?	?	?	?	?	?	?	?	-

*the credibility interval could not be reliably estimated because the likelihood surface was relatively flat.

Population migration rates obtained using this method are strikingly different from those calculated using classic estimators of gene flow. In fact, *2Nm *values between species pairs are in general very low, close to zero. Most of these values are at the lower limit of resolution because the obtained migration parameter estimates (*m1 *and *m2*) correspond to the first bin of the surveyed parameter space. However, non-zero estimates of gene flow were obtained between eight species pairs. The highest population migration rates were documented from *P. bocagei *into *P. hispanica *type 1A. A relatively high (*2Nm *> 1) migration rate was also detected from *P. hispanica *"Galera" type to *P. hispanica *sensu stricto and from the latter to *P. vaucheri*; lower but still non-zero rates were also observed from *P. hispanica *type 1B into *P. carbonelli*, from *P. hispanica *sensu stricto to *P. hispanica *type 1B, from *P. hispanica *type 3 into *P. vaucheri*, from *P. hispanica *Tunisian type to Jebel Sirwah type, and from *P. muralis *to *P. hispanica *type 3. Curiously, all these cases involve instances of asymmetrical gene flow.

In preliminary analyses, very high levels were also suspected to occur from *P. hispanica *sensu stricto into *P. hispanica *type 3, although estimates were not completely reliable because migration curves from both runs showed two peaks: one, corresponding to the maximum likelihood estimate, reflecting high levels of migration, and a smaller peak close to zero. That is, the program could not confidently choose between the hypotheses of high or zero gene flow. To solve this situation, we performed a second set of runs with a narrower prior distribution for divergence time (based on the clear peak obtained in the first runs). These analyses yielded unimodal curves for all parameters, suggesting virtually zero gene flow between the two forms.

Of these eight cases of non-zero migration rates, only three were found to be significantly different from zero (*P *< 0.05) according to a likelihood ratio test: from *P. bocagei *into *P. hispanica *type 1A, from *P. hispanica *"Galera" type into *P. hispanica *sensu stricto and from the latter to *P. vaucheri*.

Estimates of relative divergence times range from 1.64 between *P. hispanica *sensu stricto and *P. hispanica *type 3, to 3.83 between *P. carbonelli *and *P. hispanica *type 2.

## Discussion

### Lack of population structure in nuclear genealogies

The most striking result emerging from this study is a complete lack of monophyly at nuclear genes in Iberian and North African species of *Podarcis*. Both nuclear genealogies fail to detect obvious population structure that relates to that observed in mitochondrial DNA. In fact, there is a single common pattern emerging from the study of mtDNA, allozymes and both the nuclear genealogies, which is a close relationshp between *P. hispanica *from Tunisia and Jebel Sirwah.

This situation of polyphyly in nuclear genealogies relative to mitochondrial data is not uncommon (see for example [27-29], amongst many others, for similar results). At first glance, this discordance could suggest that mitochondrial DNA-defined species of Iberian and North African *Podarcis *do not correspond to true evolutionary entities and that this differentiation is the result of stochastic or deterministic effects acting only on the mitochondrial genome. In fact, it has been demonstrated that quite deep phylogeographic breaks in single gene genealogies may appear in the absence of historical barriers to gene flow simply because of stochastic lineage sorting [30,31]. It has also been shown that mitochondrial DNA divergence may not be necessarily corroborated by nuclear divergence because of selection acting on mitochondrial DNA [32]. However, there is a remarkable degree of concordance between the units defined based on mitochondrial DNA [12-15] and those observed by morphological analyses [19-21] and multilocus nuclear data [16-18]. It is therefore unlikely that subdivision within Iberian and North African *Podarcis *is nonexistent. Why, then, do nuclear genealogies apparently not support the partitions observed in mtDNA and, in particular, in allozyme data?

A simple explanation could be a low level of diversity and consequently of resolution in estimates of relationships. However, monophyly of *Podarcis *mtDNA lineages has been observed even in mitochondrial genes evolving at a very slow rate (such as the control region data set analysed by Pinho *et al. *[15], for which ~14.5% of the positions are variable (compared to ~22% in *6-Pgdint7 *and ~19.7% in *β-fibint7*); also, if a lack of resolution was responsible for the polyphyly of mtDNA groups, then we would expect that no significant differences would be observed in tree likelihoods with and without enforced species' monophyly. Tests involving topological constraints, however, demonstrated that this is not the case.

### Distinguishing between incomplete lineage sorting of ancestral polymorphism and gene flow

Given that lack of informative variation is not a satisfactory explanation for the discrepant results observed between mtDNA and nuclear genealogies, we are left with two possible scenarios, which are not mutually exclusive: a) incomplete lineage sorting of ancestral polymorphism and b) gene flow, particularly male-biased.

In order to investigate whether, despite their polyphyly, species of wall lizards are differentiated with respect to nuclear genealogies, it becomes critical to discriminate between the influences of these two scenarios in shaping the patterns of allele sharing. In order to do so, we estimated levels of gene flow based on nuclear genealogies. Our data strongly suggest that, although gene flow may be important between some species pairs, the polyphyletic pattern mostly derives from the incomplete lineage sorting of ancestral polymorphism. This is a likely scenario because nuclear genes take on average four-times as much time to reach monophyly than mitochondrial DNA does [33]. Therefore, when differentiation is recent, there is a distinct possibility that mitochondrial lineages may not be monophyletic with respect to nuclear genealogies. Three major lines of evidence support our conclusion of a greater effect of incomplete lineage sorting:

#### Comparison with allozyme variation

If gene flow was the main cause for the non-monophyly of species when considering nuclear gene variation, then allozyme data would also fail to observe any differentiation. This is certainly not the case in Iberian and North African *Podarcis*. A recent study documented not only that species are in general highly differentiated and monophyletic when considering a population tree, but also that most species can be individualized as discrete clusters by taking into account individual multilocus genotypes [18]. There are nevertheless exceptions with this regard, since some species pairs could not be distinguished, but even if these exceptions resulted solely from introgression, the correspondent pattern in nuclear genealogies would be the sharing of alleles between these particular hybridizing forms and not others, which is not verified.

#### Relationships between allele age and transpecificity

**Figure 3:**
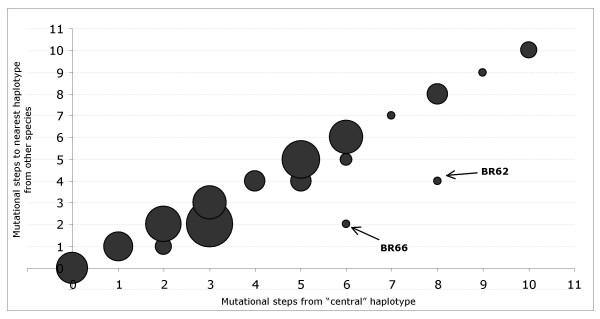
**Relationship between allele ancestrality and trans-specific proximity for locus *β-fibint7***. Ancestrality was measured as the distance, in number of mutations, to haplotype BR25, while trans-species allele proximity was measured as the distance to the closest-related allele found in a different species. The area of each circle is proportional to the allele frequency. Alleles belonging to *P. muralis *(including the supposedly introgressed allele found in *P. hispanica *type 3) were excluded. Other putatively introgressed alleles are highlighted.

A second line of evidence suggesting a major role for incomplete lineage sorting relies on the simple observation of patterns of allele relationships, particularly on the *β-fibint7 *data set. If gene flow was the main cause for the observed species polyphyly, we would expect that both ancestral (alleles that have a central position in the haplotype network) and derived alleles were transpecific or closely related to alleles found in other species. However, we do not detect this pattern; instead, we find that putatively older alleles are more widespread among species than alleles placed as tips, which are likely to have arisen after the species were separated. In order to visualize this trend, we plotted for the *β-fibint7 *data set the number of mutations separating a given haplotype from haplotype BR25, placed in the centre of the network, against the number of mutations separating that particular haplotype from the nearest haplotype found in another species (Fig. 3). The ancestral nature of haplotype BR25 was confirmed using an alternative method of haplotype network construction [34], which pinpoints the haplotype that is most probably the ancestral within a network. Although Figure 3 is a very rough representation, it clearly illustrates the relationship between allele "ancestrality" and trans-species proximity. Exceptions to this "rule" therefore constitute the most obvious cases of gene flow, as documented by the outlier position of the haplotypes BR62 and BR66 (putatively introgressed from *P. hispanica *Galera type into *P. hispanica *sensu stricto). The results are not as clear for *6-Pgdint7*, both because a central haplotype cannot be pinpointed with confidence and because this gene shows more segregation between species groups. Nevertheless, there is still a positive correlation between both measures (results not shown).

#### Non-equilibrium estimates of gene flow between species

Inferences based on coalescent simulations in a divergence with gene flow framework [11] suggest low levels of gene flow among forms, since we estimated virtually zero levels of historical gene flow between all but eight species pairs, of which only three cases were actually found to be significantly different from zero, Moreover, only one of these corresponds to important levels of gene flow since divergence.

Taken together, these results illustrate that the polyphyletic pattern probably arises as a consequence of the incomplete lineage sorting of ancestral polymorphism, and that, even though they are non-monophyletic, Iberian and North African wall lizard species are indeed overall differentiated with respect to nuclear genealogies.

### Differences between equilibrium and non-equilibrium estimates of migration rates

This study highlights how different estimates of gene flow provide different pictures of the evolutionary dynamics of a species group. On one hand, according to *F*_*ST*_-based estimates of *Nm*, most species pairs have exchanged genes since they began to diverge; indeed, only a few comparisons suggest the absence of gene flow (Table 2). Based on these estimates, we would be led to think that gene flow is a strong evolutionary force shaping genetic variability between species of *Podarcis*. On the other hand, gene flow estimates based on an isolation with migration model revealed only a few cases of clear genetic exchange between species. These results need to be interpreted with caution because the isolation with migration model that was fitted to our data does not directly apply to a multi-species scenario. However, as discussed above, these results are inline with inferences based on allozyme data and with the patterns of allele sharing or proximity, suggesting that gene flow, although existent, has probably had a minor role in shaping these lizards' evolution.

These differences between both classes of estimators are not completely unexpected. Wright's [7] island model of migration, on which the relationship between *F*_*ST *_and *Nm *is based, is indeed subject to numerous assumptions, of which several (if not all) are hardly met in most biological systems. One assumption of particular relevance to this study is that of equilibrium between migration and drift, which is violated in populations that have recently become completely isolated. In such cases low *F*_*ST *_does not necessarily translate into high levels of gene flow. This is a particular concern when estimating gene flow between species rather than populations, since in these cases effective population size are typically high and migration rates low, which implies that equilibrium conditions will take a very long time to be reached [9].

The pitfalls of gene flow estimates based on *F*_*ST *_have been widely debated in the literature [8,9,35]. However, only a few studies have actually demonstrated the influence of these limitations in the inference of migration rates in non-equilibrium conditions [36,37]. The present study therefore provides an additional warning against the misuse of equilibrium-based measures of gene flow on recently-diverged evolutionary groups.

Noteworthy, this warning is valid not only for *F*_*ST*_-based estimates but for any method that assumes equilibrium migration, even if other problems inherent to *F*_*ST *_are minimized [38].

### Evidence for historical gene flow among *Podarcis *species

Although our results argue in favour of a major role for incomplete lineage sorting in shaping the observed polyphyletic patterns in nuclear gene genealogies, we identified a few species pairs that have probably been exchanging genes.

Very high levels of admixture were detected from *P. bocagei *to *P. hispanica *type 1A. Albeit lower, still significant levels were detected from *P. hispanica *"Galera" type to *P. hispanica *sensu stricto and from *P. hispanica *sensu stricto into *P. vaucheri*. Excluding the latter, these cases do not constitute a surprise since suggestions of hybridization and introgression between these species pairs have been described before. Concerning *P. bocagei *and *P. hispanica *type 1A, these were based on individual multilocus genotype clustering [18] and the observation of interspecific matings in nature (R. Ribeiro and M. A. Carretero, pers. comm.). Similarly, gene flow between *P. hispanica *"Galera" type and *P. hispanica *sensu stricto had also been reported based on discordance between mitochondrial and morphological variation, with some additional evidence from nuclear data [23].

Our results suggest that gene flow seems to have been more frequent between forms that are sympatric at least in part of their distribution (*P. bocagei/P. hispanica *type 1A; *P. hispanica *"Galera"/*P. hispanica *sensu stricto), which is not totally unexpected. However, we also find strong support for gene flow events between *P. vaucheri *and *P. hispanica *sensu stricto. These two species still have poorly studied distribution limits, so it is yet unknown if they establish contact zones or if they are completely allopatric. Nevertheless, in theory present contact should not be a prerequisite for gene exchange; given that species have most likely suffered repeated pulses of range expansions and contractions due to geological or climatic events, presently allopatric pairs of species may have met at some point in their past and then hybridized.

Besides spatial considerations, other results regarding introgression between species are noteworthy. First, in the case of *P. bocagei *and *P. hispanica *type 1A, the inferred levels of admixture were surprisingly high, above the values considered as thresholds for the maintenance of genetic differentiation [7]. However, the two species are sympatric and nevertheless maintain their morphological identifiability. It has also been shown that males discriminate conspecific from heterospecific females based on chemical stimuli, which suggests at least some degree of assortative mating [39]. Furthermore, the two species correspond to clearly distinct genetic clusters as shown by almost perfect assignment using individual allozyme multilocus genotype clustering approaches [18]. It therefore seems that the present estimates of gene flow between these two species are slightly inflated; this unexpected outcome could result from our limited sampling and therefore needs further clarification.

Second, we should discuss in more detail the situation of *P. hispanica *sensu stricto. It has been suggested that the divergent mitochondrial clade that we refer to as *P. hispanica *sensu stricto is a "ghost" lineage; in other words, this divergent mtDNA clade would be an extant signature of a nowadays extinct species whose mtDNA survived in the nuclear background of other species inhabiting its former area of distribution (namely *P. hispanica *Galera type and *P. hispanica *type 3) [23]. Similar scenarios of mtDNA persistence through interspecific capture and nuclear dilution have in fact been shown to occur in several other cases [40,41], so this hypothesis would not be completely farfetched. However, our results do not agree with this scenario. On one hand, we find that significant amounts of gene flow have indeed occurred between the mtDNA-defined *P. hispanica *sensu stricto and *P. hispanica *Galera type. However, these levels do not seem to have been large enough for the two forms to stand out as undifferentiated. The same situation is suggested by analyses of allozyme variation, which report high levels of differentiation and a complete lack of gene flow between nearby interspecific populations of these two taxa [18]. On the other hand, with respect to *P. hispanica *type 3, although this was unclear in preliminary runs, IM returned very low population migration rates in both directions. This therefore contrasts with analyses of allozyme data, in which these two forms were found to be non-diagnosable [18]. Because data with respect to the nuclear differentiation of mitochondrial DNA lineages from eastern Iberian Peninsula thus remains controversial, this subject clearly needs further assessment.

Third, although migration from *P. muralis *to *P. hispanica *type 3 was found to be non significant according to the test proposed by Nielsen and Wakeley [10], there is strong evidence suggesting that gene flow may have occurred between these two species. In fact, an allele separated by a single mutation from those typical of *P. muralis *alleles, which in turn are distinct by several mutations from the ingroup, was detected in an individual with *P. hispanica *type 3 mtDNA. For this to correspond to an allele shared by incomplete lineage sorting, it would imply that a single mutation had occurred in either of the lineages since the divergence of these two species (over 10 million years ago [42]), which is unlikely. We therefore believe this to be a false negative for the occurrence of migration, which the conservative significance test is prone to produce [10]. Although the confirmation of this result requires further investigation, this may imply that species of *Podarcis *take a long time of divergence to acquire complete reproductive isolation.

Nevertheless, this study should not be taken as a definitive assessment of gene flow among *Podarcis *species. First, because other false negatives may exist among the species pairs in which IM identified non-trivial migration rates. Second, and most importantly, because our limited sample size does not allow pinpointing with complete certainty which species have exchanged genes and which have not. We may infer from this work that historical gene flow among Iberian and North African *Podarcis *has been low, yet existent; however, a detailed analysis of the patterns of genetic exchange across species will require the detection and thorough characterization of contact zones.

### Implications regarding the evolutionary patterns of Iberian and North African *Podarcis*

A primary observation that can be drawn from this work is that single nuclear genealogies do not reflect a branching pattern related to putative isolation in allopatry that led to the formation of the observed species, mainly because of shared ancestral polymorphism. Hudson and Coyne [43] estimated that, assuming that mutation and drift are the only evolutionary forces operating, reciprocal monophyly should be attained at ~95% of nuclear loci 9N to 12N generations after the population split. It is noteworthy that the minimum separation time between the various lineages has been estimated to be around 5 million years (i.e ~2 million generations), based on mtDNA phylogenetic analyses. Assuming that these estimates are correct, the fact that mtDNA lineages are not reciprocally monophyletic at the nuclear level suggest that the majority, if not all, of extant Iberian and North African *Podarcis *lineages were able to maintain a relatively high historical effective population size throughout their presumably eventful history, which allowed sorting of mtDNA lineages to monophyly without the corresponding pattern in nuclear genes. We hypothesise that larger effective population sizes in *Podarcis *might explain the contrasting patterns of subdivision observed at nuclear loci between wall lizards and, for example, the Iberian newt *Lissotriton boscai*. This species complex is characterized by similarly old or even younger mtDNA splits [44], but in this case the various groups have reached almost complete reciprocal monophyly at the *β-fibint7 *locus [45].

Pinho *et al. *[18] suggested that contrasting evolutionary scenarios inferred for the evolution of the genus on the basis of mitochondrial vs. nuclear markers (i.e. step-by-step speciation vs. radiation) could be accommodated taking into account the different effective population sizes that characterize both classes of markers. Indeed, speciation may have occurred at a rate fast enough so that nuclear gene genealogies are uninformative regarding species relationships but mitochondrial genealogies are not. In fact, although multi-species clades in Pinho *et al.*'s [15] mitochondrial phylogenetic assessment of relationships are supported by high bootstrap values, internal branches are short, which is consistent with a fast speciation rate. Accordingly, our estimates of divergence time between mtDNA-defined lineages on the basis of nuclear markers do not correlate to mitochondrial DNA branching patterns; for example, the largest time since divergence was inferred for the mitochondrial sister pair *P. carbonelli*/*P. hispanica *type 2.

An intriguing characteristic of both nuclear gene genealogies isthe detection of a skew towards rare alleles, as suggested by largely negative and significant Tajima's D. Although such a multilocus pattern can usually be interpreted as a signature of demographic growth, it has been shown that the same patterns may also arise as a consequence of fine-scale population structure, which is translated as a bias into sampling schemes such as ours, in which few individuals from multiple populations are analysed [46].

### A gradual view of speciation

With the analyses of nuclear genealogies in *Podarcis *wall lizards from the Iberian Peninsula and North Africa, we have provided compelling evidence for general historical reproductive isolation among distinct lineages despite their non reciprocal monophyly. These results are therefore in accordance with emergent morphological data and previous reports of mitochondrial and nuclear gene variation suggesting that these forms may correspond to different, almost completely reproductively isolated species. We have also provided evidence for the existence of limited to important levels of gene flow among a few species pairs, also in accordance with previously published data. Although the effect that such admixture processes may have in species differentiation remains to be completely acknowledged, preliminary work on the contact zone between *P. bocagei *and *P. carbonelli *suggests spatially-restricted genetic exchange and a bimodal hybrid zone concordant with the existence of strong barriers to gene flow [47].

The lack of reciprocal monophyly and the permeability to gene flow implies that wall lizard lineages will fail to be considered good species in the light of most species concepts. Nevertheless, it appears that most forms remain differentiated despite the merging influence of gene flow. Our results in *Podarcis *wall lizards therefore add to recent evidence stemming from the study of other incipient species groups (such as heliconiine butterflies [48] for example) illustrating that the acquisition of reproductive isolation is a gradual process and contributing to the establishment of a "species with fuzzy borders" paradigm.

*Podarcis *wall lizards in the Iberian Peninsula and North Africa present a wide variety of distribution patterns, including sympatric, parapatric and allopatric forms and will therefore constitute an excellent model for testing hypotheses related to the origin and maintenance of reproductive isolation in closely related species.

## Conclusion

Along with a growing body of work focusing on recent species complexes, this study supports a progressive view of speciation, according to which species may persist as differentiated despite remaining porous to genetic exchange and having poorly-defined genetic boundaries for a very long period of time after divergence. Such recent species complexes often have not yet achieved equilibrium between migration and drift, a condition which will strongly influence the outcome of studies aiming at quantifying gene flow if recent divergence is not taken into account.

## Methods

### Sampling and DNA extraction

Currently, 11 evolutionary units have been identified using mitochondrial DNA within the Iberian Peninsula and North Africa clade of *Podarcis*. The species used here as an outgroup, *P. muralis*, is phylogenetically distinct from the other forms [42,49] and has a wide distribution range throughout Europe, including Northern Iberian Peninsula. Our sampling scheme was designed to incorporate a minimum of five individuals per species, morphotype or mtDNA lineage (Table 5). The majority of individuals were preliminarily identified and assigned to an evolutionary group using morphological criteria. Because some mtDNA lineages have not been studied in detail morphologically and others that have are indistinguishable, our final assignment was based on mitochondrial DNA sequencing (see below). We will, from here on, refer to the defined groups as "species" for a matter of simplicity. It should be noted, however, that the specific status of some of these groups still remains to be evaluated. The sampling scheme was as representative of the species distributions as possible. In total, 78 individuals were analysed. The geographical locations of the samples, as well as a preliminary distribution map of mitochondrial lineages are shown in Figure 1. Samples consisted of a small tail clip, obtained taking advantage of the lizard's natural tail autotomy capacity. All lizards were released after sample collection. Samples were stored in ethanol or frozen at -80°C prior to DNA extraction, which was accomplished following standard protocols [50]. All mitochondrial DNA sequences from *P. bocagei*, *P. carbonelli *and *P. vaucheri *individuals, as well as several individuals from other species (identified in Table 5) were generated for previous studies on mitochondrial variation [15,51].

**Table 5:** Samples analysed in this study and corresponding nuclear gene genotypes. For the geographical origin of samples, see figure 1. Alleles correspond to those in figure 2. * indicates ND4 sequence previously published.

Species/MtDNA lineage	Sample Code	Locality	Country	Genotype	
				
				*6-Pgdint7*	*β-fibint7*
*P. bocagei*	Mad11*	Madalena	Portugal	PR1–PR2	BR1–BR2
	Vair12*	Vairão	Portugal	PR2-PR2	BR1–BR3
	Cor6*	Coruña	Spain	PR1-PR1	BR1-BR1
	M6*	Montesinho	Portugal	PR1-PR1	BR1–BR4
	BTA5*	Tanes	Spain	PR1–PR2	BR1-BR1
*P. carbonelli*	MC3*	Monte Clérigo	Portugal	PR3-PR3	BR5-BR5
	PR3*	Playa del Rompeculos	Spain	PR5-PR5	BR5–BR8
	A11*	Aveiro	Portugal	PR3–PR7	BR6–BR9
	SPM2*	S. Pedro de Moel	Portugal	PR6-PR6	BR7-BR7
	LA7*	La Alberca	Spain	PR3–PR4	BR10–BR11
*P. vaucheri*	LB2*	La Barrosa	Spain	PR37–PR40	-
	LB4*	La Barrosa	Spain	PR38–PR39	BR45–BR47
	LB5*	La Barrosa	Spain	-	BR46–BR47
	LB7*	La Barrosa	Spain	PR37–PR38	-
	BT6*	Bab Taza	Morocco	PR41–PR42	BR25-BR25
	Mis3*	Mischliffen	Morocco	PR41–PR42	BR48–BR49
	Ouk7*	Oukaimeden	Morocco	PR41–PR43	BR49-BR49
*P. hispanica *type 1A	Mon1	Montesinho	Portugal	PR9-PR9	BR12–BR25
	Mon2	Montesinho	Portugal	PR8–PR10	BR13–BR24
	Anc2	Los Ancares	Spain	PR8-PR8	BR15–BR18
	FT12	Tua	Portugal	-	BR21–BR23
	PG2	Atlantic Islands	Spain	PR1-PR1	BR18-BR18
	Pen2	Pendilhe	Portugal	PR11–PR12	BR17–BR26
	Pen8	Pendilhe	Portugal	PR1–PR8	BR14–BR25
	Rua1*	Vila de Rua	Portugal	-	BR18–BR22
*P. hispanica *type 1B	Trj1*	Trujillo	Spain	PR20–PR22	BR27–BR30
	Oro1*	Oropesa	Spain	PR21-PR21	BR29–BR31
	Vil3	Villacastin	Spain	-	BR20-BR20
	Vil8	Villacastin	Spain	PR17–PR20	BR19–BR20
	GuaI1	Guadarrama	Spain	PR18–PR19	BR20-BR20
	HLA1	La Alberca	Spain	PR13–PR14	BR16–BR28
*P. hispanica *type 2	CV1	Castelo de Vide	Portugal	PR23–PR24	BR32–BR33
	SM1	S. Mamede	Portugal	PR24–PR25	BR34-BR34
	Ev4	Évora	Portugal	PR27–PR29	BR35-BR35
	Mad1	Madrid	Spain	PR26–PR28	-
	Mad2*	Madrid	Spain	PR15-PR15	BR36-BR36
	And9	Saucedilla	Spain	-	BR38–BR39
	And10*	Benatae	Spain	PR26–PR27	-
	CR1	Castaño de Robledo	Spain	PR16–PR30	BR37-BR37
*P. hispanica *sensu stricto	Cue2	Cuenca	Spain	-	BR43–BR44
	Mot1*	Motilla del Palancar	Spain	PR31–PR33	-
	Pod12*	Granada	Spain	PR3–PR34	BR41-BR41
	And8*	Puebla de D. Fadrique	Spain	PR32–PR34	BR40–BR43
	SN2	Sierra Nevada	Spain	PR33–PR34	BR25–BR42
	SN10	Sierra Nevada	Spain	PR35-PR35	BR25-BR25
	SN11	Sierra Nevada	Spain	PR36-PR36	BR25–BR42
	BEV7337	Sta. Maria de Nieva	Spain	PR56–PR58	BR62–BR66
*P. hispanica *type 3	Barc5	Barcelona	Spain	PR33–PR50	BR56–BR57
	Bur2*	Burgos	Spain	PR33-PR33	BR25-BR25
	Get1	Getaria	Spain	PR52-PR52	BR70-BR70
	Med1*	Medinaceli	Spain	PR3–PR51	BR58–BR59
	Pht1	Tarragona	Spain	PR53–PR54	BR60-BR60
*P. hispanica *Galera	Gal1x	Galera	Spain	PR55–PR57	BR64–BR65
	Gal1	Galera	Spain	-	BR63-BR63
	Gal3*	Galera	Spain	PR55-PR55	-
	Gal3x	Galera	Spain	PR55–PR56	BR67-BR67
	Gal5x	Galera	Spain	-	BR65–BR67
	BEV7353	Puebla de D. Fadrique	Spain	PR55–PR56	BR68–BR69
	Gal2	Galera	Spain	PR55-PR55	-
	Gal7x	Galera	Spain	PR55-PR55	BR61–BR67
*P. hispanica *Jebel Sirwah	JS1*	Jebel Sirwah	Morocco	PR44–PR46	BR50–BR51
	JS2	Jebel Sirwah	Morocco	PR45–PR46	BR51-BR51
	JS3	Jebel Sirwah	Morocco	PR45–PR46	BR51-BR51
	JS6*	Jebel Sirwah	Morocco	PR44–PR46	BR51-BR51
	PH184	Jebel Sirwah	Morocco	PR45–PR46	BR52–BR53
	PH186	Jebel Sirwah	Morocco	PR44-PR44	BR51-BR51
*P. hispanica *Tunisia	OK1*	Oued Kébir	Tunisia	PR44–PR49	BR54-BR54
	OK8	Oued Kébir	Tunisia	PR44–PR49	BR55-BR55
	OK11	Oued Kébir	Tunisia	PR47–PR48	BR54-BR54
	LK5	Le Kef	Tunisia	PR49-PR49	BR55-BR55
	LK6*	Le Kef	Tunisia	PR44–PR49	BR55-BR55
*P. muralis*	MTA1*	Tanes	Spain	-	BR71-BR71
	MTA2	Tanes	Spain	-	BR71–BR72
	MTA3	Tanes	Spain	PR59-PR59	BR71-BR71
	MTA4	Tanes	Spain	PR60-PR60	BR71-BR71
	Gua1*	Guadarrama	Spain	PR61-PR61	-
	Gua2	Guadarrama	Spain	PR61-PR61	BR71-BR71
	Gua13	Guadarrama	Spain	PR61-PR61	BR71-BR71

### DNA amplification, sequencing and haplotype determination

We generated sequences from two nuclear genes: *β*-fibrinogen intron 7 (*β-fibint7*) and 6-phosphogluconic acid dehydrogenase intron 7 (*6-Pgdint7*). To establish a correct assignment of all individuals to a mitochondrial DNA lineage, we further sequenced a mitochondrial fragment comprising the 3' end of the NADH dehydrogenase subunit 4 gene and adjacent tRNAs (from here on referred to as ND4). A list of the primers that were developed for amplification of the three studied genes is given in Table 6.

**Table 6:** Primers developed for this study

Gene region	Primer name	Primer sequence
ND4	GalND4F	5'-TGC TAA AAC TAG GTG GCT ATG GCT TAA TCC GCA TC-3'
	GalND4R	5'-TCT CGA GTG TGG GTG GGA GGA AGG AGT CGA AT-3'
*6-Pgdint7*	PgdP7F	5'-GAC ATG CAG CTG ATC TGT GAG GCC-3'
	PgdP8R	5'-GAG TCC AGC TCA GTC TTA TTC CAC-3'
	PGD500	5'-CAT TTG CTC TTA AGA AAA TAG GAA G-3'
*β-fibint7*	BF8	5'-CAC CAC CGT CTT CTT TGG AAC ACT G-3'
	BfibR	5'-CAG GGA GAG CTA CTT TTG ATT AGA C-3'

Amplification and sequencing of the ND4 gene was accomplished using primers ND4 and Leu [52] and followed the conditions described by Pinho *et al. *[15]. In this work the authors reported the existence of a nuclear pseudogene of ND4 in the *P. hispanica *morphotype from the population of Galera that was amplified instead of the mitochondrial fragment (see [15] for details). To avoid the amplification of this nuclear copy we developed primers targeting only the mitochondrial sequence observed in this morphotype (primers GalND4F and GalND4R, described in Table 6). Sequences obtained through the use of these primers were apparently single-copy and similar to the mitochondrial sequence inferred by Pinho *et al. *[15].

At a preliminary stage, amplification of the *β-fibint7 *gene was accomplished using primers FIB-B17U and FIB-B17L [53] under the conditions described by Godinho *et al. *[54], but lowering the annealing temperature to 50°C. However, because amplification and sequencing success was low with these primers, we tried several other combinations of primers, both already published and newly designed for this study. The definitive amplification and sequencing of this gene was accomplished using primer BFXF described in Sequeira *et al. *[55], as well as a new primer (BF8) designed considering the alignment of sequences from *Podarcis *with those from several *Lacerta *species [54]. Amplification was carried out in 20 μL volumes, containing 2 μL 10× reaction buffer (Ecogen), 2 mM MgCl_2_, 0.4 mM each dNTP, 0.2 μM each primer, 1 unit of *Ecotaq *DNA polymerase (Ecogen) and approximately 100 ng of genomic DNA. Amplification conditions consisted of a pre-denaturing step of 3 min at 92°C followed by 40 cycles of a denaturing step of 30 s at 92°C, annealing at 53°C for 30 s and extension at 72°C for 90 s. The final extension was accomplished at 72°C for 5 min. This protocol failed to amplify some of the samples, including all *P. hispanica *with the Galera mtDNA type. We therefore designed an internal primer, BfibR, which was used together with primer BF8 under the same conditions described above.

The locus *6-Pgdint7 *was chosen for this work based on very high polymorphism levels observed for the studied lizards in the allozyme encoded by the *6-Pgd *gene [16-18]. We began by aligning mRNA sequences for several organisms (*Homo sapiens*, accession number BC000368, *Mus musculus*, accession number BC011329, *Danio rerio*, accession number AY391449 and *Lasaea australis*, accession number AF345495). Highly conserved regions situated on human exons 7 and 8 were targeted to design multiple degenerate primers for exon-primed, intron-crossing (EPIC) PCR [56]. Several combinations of these were tried on a limited number of *Podarcis *samples under gradients of annealing temperatures and MgCl_2 _concentrations. The beginning, supposedly exonic portions of the amplified fragments revealed a high degree of homology with the mRNA sequences used to construct the initial alignment, suggesting that the sequenced genomic portion was indeed intron 7 of the *6-Pgd *gene. New primers, named PgdP7F and PgdP8R, specific for *Podarcis*, were then designed in the exons and allowed successful amplification of this gene region. Optimal amplification was achieved using the same conditions described above for *β-fibint7*, with the exception that some samples required annealing temperatures of 60°C. An internal reverse primer, PGD500, was also designed for sequencing purposes.

The PCR products were enzymatically purified and sequenced using the ABI Prism BigDye Terminator Cycle sequencing protocol in an ABI Prism 310 automated sequencer (Applied Biosystems) with the same primers used for amplification.

We used two approaches to resolve the haplotype phase of nuclear DNA sequences. First, for sequences that were heterozygous for insertions or deletions, we used the method described by Flot *et al. *[57]. Second, we used the Bayesian algorithm implemented in the PHASE software [58], using known phases of haplotypes determined by the previous method. Several individuals were sequenced for which the haplotype phase could not be completely determined; these individuals were not included in subsequent analyses and for simplicity are not represented in Table 5 and Figure 1. Sequences were aligned manually using BIOEDIT v. 7.0.5.2 [59]. For nuclear genes, each allele was coded with the name of the individual carrying it following by the letters A or B.

### Analytical methods

#### Mitochondrial DNA phylogeny

We used an alignment of 536 bp of the ND4 gene to carry out phylogenetic analyses under a maximum likelihood approach using GARLI version 0.95 [60,61]. This software allows simultaneous estimates of tree topology, branch lengths and model parameters under the general-time-reversible model of sequence evolution.

#### Nuclear gene genealogies

To illustrate relationships between haplotypes, a median-joining network [62] was built using NETWORK [63]. We preferred this method of representing genealogies rather than phylogenetic trees because the levels of polymorphism observed at both nuclear genes were much lower than those observed in mitochondrial DNA. For these situations, haplotype networks constitute a better way of representing relationships [64]. Because multiple-base insertions or deletions are likely to have resulted from a single evolutionary step, we pruned the data in order to leave only the first base of the indel. We chose this approach rather than completely removing indels because this would significantly reduce the number of polymorphic sites used to build the network and disregard much of the information contained in the data sets. Nevertheless, some segregating positions located within areas of indels were removed by this pruning method.

In order to test alternative hypotheses (see Results) relative to the topology of the nuclear gene genealogies, we also searched for ML trees that better represented the phylogeny of the observed haplotypes using GARLI. Under the model of sequence evolution derived from these analyses, we performed new ML searches enforcing a particular topology using PAUP* 4.0b10 [65] and compared the resulting trees' likelihoods with those obtained from unconstrained analyses using Shimodaira-Hasegawa tests [26] under the RELL approximation with 1000 bootstrap replicates. This step was conducted using the full data sets (i.e. without removing indels from the alignment).

#### DNA sequence polymorphism, population subdivision and recombination

All the following analyses were performed using reduced data sets from which all indels were eliminated. We calculated summary diversity statistics (number of haplotypes, number of segregating sites, nucleotide diversity, *π*, and the population mutation parameter θ [66] for the three data sets and separately for each partition. We also computed Tajima's D [67] to test for non-neutral evolution of the analysed data-sets. We also computed values of Hudson *et al.*'s [68]*F*_*ST *_between all species pairs. These analyses were conducted in DNASP [69]. To evaluate the possibility of recombination in the nuclear genes, we computed Hudson and Kaplan's [24] Rm statistic (representing the minimum number of recombination events) using DNASP. Because this statistic is likely to be highly affected by homoplasy, we also used the software PHIPACK [70] to test for recombination using the pairwise homoplasy index (Φ_*w *_statistic) of Bruen et al. [25]. This statistic is a powerful detector of recombination and has been shown by simulation studies to be less sensitive to the effects of mutation rate correlation than other available statistics, which are prone to falsely infer recombination when levels of recurrent mutation are high [25]. PHIPACK provides *P*-values for the acceptance of the null hypothesis of no recombination. We used the two available options to estimate such *P*-values: an analytical approach and a permutation test (1000 permutations).

#### Estimation of migration rates between species

We used two different approaches to assess levels of gene flow between forms. First, *Nm *values were inferred from *F*_ST _according to Wright's [7] island model of population structure using the expression *F*_ST _≈ (1+2*Nm*) for mitochondrial DNA and *F*_ST _≈ 1/(1+4*Nm*) for nuclear autosomal loci. Traditional, *F*_ST_-based methods of estimating *Nm *make many unrealistic assumptions, among which is the equilibrium between drift and migration. Species that have recently diverged often share much of their genetic variation not only due to gene flow but also because of retention of ancestral polymorphism. In these cases, obtaining realistic estimates of migration between populations is challenging. Nielsen and Wakeley [10] developed a model which takes into account population divergence and gene flow in the same framework, thus being appropriate to disentangle between the relative effects of isolation and migration in shaping the patterns of variation among diverging species. This model was extended to incorporate multilocus data by Hey and Nielsen [11], as implemented in the IM computer program, which works under a two-population model and uses a Markov Chain Monte Carlo approach to estimate six parameters scaled by the neutral mutation rate: effective population sizes for both extant populations and their ancestor, time since divergence and migration rates on both directions. This model was developed for a simple two-population scenario and therefore does not directly apply to the complexity inherent to divergence within Iberian and North African *Podarcis*, in which eleven mitochondrial DNA lineages have been described. Nevertheless, because no applications of this model have been developed to deal with more than two populations, we performed pairwise analyses to assess historical migration rates between all species pairs. We included the outgroup, *P. muralis*, in these analyses, because preliminary inspection of sequence data for one of the studied gene regions suggested that introgression with the ingroup could have occurred (see Results). Several analyses involving more than two species have been successfully performed using IM or similar methods [29,36,71-73]. It should be kept in mind, however, that using a two population model influence from other populations might have an unpredictable effect on our estimates. In order to minimize such effect, we performed a second set of runs for comparisons involving *P. hispanica *sensu stricto and *P. hispanica *type 3, excluding alleles that were apparently introgressed from other species (see Results).

Since IM cannot accommodate gaps in DNA sequence data, we pruned all gaps and missing data from the data sets. An assumption made by IM is no recombination; because we had no evidence for recombination in our data sets using Bruen *et al*. 's [25] method (see Results), we used the complete data sets in the analyses. We used the HKY [74] model in all analyses. After several experimental runs to assess appropriate parameter settings and ensure proper mixing, IM was run for each two-species data set for 20 million steps along the Markov Chain after 1 million steps of burn-in (using the ramped heating scheme, option -bh), with 5 Metropolis-coupled chains with linear heating. For each data set, a second, smaller run with 10 million steps using the same options and a different random seed was used to confirm convergence of parameter estimates.

To investigate whether estimates of migration rates were significantly different from zero, we compared the value of the likelihood for zero migration with the maximum likelihood for this parameter, by calculating the log-likelihood ratio between the two. The distribution of the log-likelihood test statistic (minus two times the log-likelihood ratio) can be approximated taking into account the distribution of a random variable which takes the value 0 with probability 0.5 and takes on a value from a χ^2 ^distribution with 1 degree of freedom with 0.5 probability [10].

## Authors' contributions

CP carried out molecular laboratory work, analysed the data and drafted the manuscript. All authors participated in the conception and design of the study, writing and approval of the final manuscript.

## Supplementary Material

Additional file 1 Correspondence between alleles in the complete data sets and alleles in reduced data sets used to build haplotype networks. Click here for file
