# Supplementary material for: Non-equilibrium estimates of gene flow inferred from nuclear genealogies suggest that Iberian and North African wall lizards (Podarcis spp.) are an assemblage of incipient species
Source: BMC Evol Biol. 2008 Feb 26;8:63. doi: 10.1186/1471-2148-8-63 (PMC2277379; doi:10.1186/1471-2148-8-63)
Supplement: Additional file 1 — Correspondence between alleles in the complete data sets and alleles in reduced data sets used to build haplotype networks. [file 1471-2148-8-63-S1.doc]

| *6-Pgdint7* | | | |
| --- | --- | --- | --- |
| Allele | Sample | GenBank accession number | Allele in haplotype network |
| P1 | Mad11A, BTA5B, M6A, M6B, Cor6A, Cor6B, Pen8A, PG2A, PG2B | EU269595 | PR1 |
| P2 | Mad11B, Vair12A, Vair12B, BTA5A | EU269596 | PR2 |
| P3 | MC3A, MC3B, A11B, LA7A, Med1B | EU269597 | PR3 |
| P4 | LA7B | EU269598 | PR4 |
| P5 | PR3A, PR3B | EU269599 | PR5 |
| P6 | SPM2A, SPM2B | EU269600 | PR6 |
| P7 | A11A | EU269601 | PR7 |
| P8 | Anc2A, Anc2B, Mon2B, Pen8B | EU269602 | PR8 |
| P9 | Mon1A, Mon1B | EU269603 | PR9 |
| P10 | Mon2A | EU269604 | PR10 |
| P11 | Pen2A | EU269605 | PR11 |
| P12 | Pen2B | EU269606 | PR12 |
| P13 | HLA1A | EU269607 | PR13 |
| P14 | HLA1B | EU269608 | PR14 |
| P15 | Mad2A, Mad2B | EU269609 | PR15 |
| P16 | CR1A | EU269610 | PR16 |
| P17 | Vil8B | EU269611 | PR17 |
| P18 | GuaI1A | EU269612 | PR18 |
| P19 | GuaI1B | EU269613 | PR19 |
| P20 | Vil8A, Trj1A | EU269614 | PR20 |
| P21 | Oro1A, Oro1B | EU269615 | PR21 |
| P22 | Trj1B | EU269616 | PR22 |
| P23 | CV1B | EU269617 | PR23 |
| P24 | CV1A, SM1B | EU269618 | PR24 |
| P25 | SM1A | EU269619 | PR25 |
| P26 | And10B, Mad1A | EU269620 | PR26 |
| P27 | And10A, Ev4B | EU269621 | PR27 |
| P28 | Mad1B | EU269622 | PR28 |
| P29 | Ev4A | EU269623 | PR29 |
| P30 | CR1B | EU269624 | PR30 |
| P31 | Mot1A | EU269625 | PR31 |
| P32 | And8A | EU269626 | PR32 |
| P33 | Mot1B, Bur2A, Bur2B | EU269627 | PR33 |
| P34 | Pod12A | EU269628 | PR3 |
| P35 | Pod12B, SN2B, And8B | EU269629 | PR34 |
| P36 | SN2A, SN10A, SN10B | EU269630 | PR35 |
| P37 | SN11A, SN11B | EU269631 | PR36 |
| P38 | LB2A, LB7A | EU269632 | PR37 |
| P39 | LB4B, LB7B | EU269633 | PR38 |
| P40 | LB4A | EU269634 | PR39 |
| P41 | LB2B | EU269635 | PR40 |
| P42 | BT6A, Mis3A | EU269636 | PR41 |
| P43 | Ouk7B | EU269637 | PR41 |
| P44 | BT6B, Mis3B | EU269638 | PR42 |
| P45 | Ouk7A | EU269639 | PR43 |
| P46 | JS1A, JS6A, PH186A, PH186B | EU269640 | PR44 |
| P47 | JS2A, JS3B, PH184B | EU269641 | PR45 |
| P48 | JS3A, JS2B, PH184A, JS1B, JS6B | EU269642 | PR46 |
| P49 | OK11A | EU269643 | PR47 |
| P50 | OK11B | EU269644 | PR48 |
| P51 | OK1A, OK8A, LK6A | EU269645 | PR44 |
| P52 | OK1B, OK8B, LK5A, LK5B, LK6B | EU269646 | PR49 |
| P53 | Barc5A | EU269647 | PR50 |
| P54 | Barc5B | EU269648 | PR33 |
| P55 | Med1A | EU269649 | PR51 |
| P56 | Get1A, Get1B | EU269650 | PR52 |
| P57 | PhT1B | EU269651 | PR53 |
| P58 | PhT1A | EU269652 | PR54 |
| P59 | Gal3A, Gal3B, Gal3xA, BEV7353A, Gal2A, Gal2B, Gal7xA, Gal7xB, Gal1xA | EU269653 | PR55 |
| P60 | BEV7353B, BEV7337B, Gal3xB | EU269654 | PR56 |
| P61 | Gal1xB | EU269655 | PR57 |
| P62 | BEV7337A | EU269656 | PR58 |
| P63 | MTA3A, MTA3B | EU269657 | PR59 |
| P64 | MTA4A, MTA4B | EU269658 | PR60 |
| P65 | Gua1A, Gua1B, Gua2A, Gua2B, Gua13A, Gua13B | EU269659 | PR61 |

Additional file 1 – Correspondence between alleles in the complete data sets and alleles in reduced data sets used to build haplotype networks.

| *β-fibint7* | | | |
| --- | --- | --- | --- |
| Allele | Sample | GenBank accession number | Allele in haplotype network |
| B1 | Mad11A, Vair12A, BTA5A, BTA5B, M6A, Cor6A, Cor6B | EU269471 | BR1 |
| B2 | Mad11B | EU269472 | BR2 |
| B3 | Vair12B | EU269473 | BR3 |
| B4 | M6B | EU269474 | BR4 |
| B5 | MC3A, MC3B, PR3B | EU269475 | BR5 |
| B6 | A11A | EU269476 | BR6 |
| B7 | SPM2A, SPM2B | EU269477 | BR7 |
| B8 | PR3A | EU269478 | BR8 |
| B9 | A11B | EU269479 | BR9 |
| B10 | LA7A | EU269480 | BR10 |
| B11 | LA7B | EU269481 | BR11 |
| B12 | Mon1A | EU269482 | BR12 |
| B13 | Mon2A | EU269483 | BR13 |
| B14 | Pen8A | EU269484 | BR14 |
| B15 | Anc2B | EU269485 | BR15 |
| B16 | HLA1A | EU269486 | BR16 |
| B17 | Pen2A | EU269487 | BR17 |
| B18 | Anc2A | EU269488 | BR18 |
| B19 | Rua1A, PG2A, PG2B | EU269489 | BR18 |
| B20 | Vil8A | EU269490 | BR19 |
| B21 | Vil8B, Vil3A, Vil3B, GuaI1A, GuaI1B | EU269491 | BR20 |
| B22 | FT12A | EU269492 | BR21 |
| B23 | Rua1B | EU269493 | BR22 |
| B24 | FT12B | EU269494 | BR23 |
| B25 | Mon2B | EU269495 | BR24 |
| B26 | Mon1B | EU269496 | BR25 |
| B27 | Pen8B | EU269497 | BR25 |
| B28 | Pen2B | EU269498 | BR26 |
| B29 | Trj1A | EU269499 | BR27 |
| B30 | HLA1B | EU269500 | BR28 |
| B31 | Oro1A | EU269501 | BR29 |
| B32 | Trj1B | EU269502 | BR30 |
| B33 | Oro1B | EU269503 | BR31 |
| B34 | Mad1A, Mad1B | EU269504 | * |
| B35 | CV1A | EU269505 | BR32 |
| B36 | CV1B | EU269506 | BR33 |
| B37 | SM1A | EU269507 | BR34 |
| B38 | SM1B | EU269508 | BR34 |
| B39 | Ev4A, Ev4B | EU269509 | BR35 |
| B40 | Mad2A, Mad2B | EU269510 | BR36 |
| B41 | CR1A, CR1B | EU269511 | BR37 |
| B42 | And9A | EU269512 | BR38 |
| B43 | And9B | EU269513 | BR39 |
| B44 | And8A | EU269514 | BR40 |
| B45 | Pod12A, Pod12B | EU269515 | BR41 |
| B46 | SN11B, SN2A | EU269516 | BR42 |
| B47 | Cue2B, And8B | EU269517 | BR43 |
| B48 | Cue2A | EU269518 | BR44 |
| B49 | SN11A, SN2B, SN10A, SN10B | EU269519 | BR25 |
| B50 | LB4A | EU269520 | BR45 |
| B51 | LB5A | EU269521 | BR46 |
| B52 | LB5B, LB4B | EU269522 | BR47 |
| B53 | BT6A | EU269523 | BR25 |
| B54 | BT6B | EU269524 | BR25 |
| B55 | Mis3B | EU269525 | BR48 |
| B56 | Mis3A, Ouk7A, Ouk7B | EU269526 | BR49 |
| B57 | JS1B | EU269527 | BR50 |
| B58 | JS1A, JS2A, JS2B, JS3A, JS3B, JS6A, JS6B, PH186A, PH186B | EU269528 | BR51 |
| B59 | PH184A | EU269529 | BR52 |
| B60 | PH184B | EU269530 | BR53 |
| B61 | OK1A, OK1B, OK11A, OK11B | EU269531 | BR54 |
| B62 | OK8A, OK8B, LK5A, LK5B, LK6A, LK6B | EU269532 | BR55 |
| B63 | Barc5A | EU269533 | BR56 |
| B64 | Barc5B | EU269534 | BR57 |
| B65 | Bur2A, Bur2B | EU269535 | BR25 |
| B66 | Med1A | EU269536 | BR58 |
| B67 | Med1B | EU269537 | BR59 |
| B68 | PhT1A, PhT1B | EU269538 | BR60 |
| B69 | Gal7xA | EU269539 | BR61 |
| B70 | BEV7337B | EU269540 | BR62 |
| B71 | Gal1A, Gal1B | EU269541 | BR63 |
| B72 | Gal1xB | EU269542 | BR64 |
| B73 | Gal1xA, Gal5xA | EU269543 | BR65 |
| B74 | BEV7337A | EU269544 | BR66 |
| B75 | Gal3xA, Gal3xB, Gal5xB, Gal7xB | EU269545 | BR67 |
| B76 | BEV7353A | EU269546 | BR68 |
| B77 | BEV7353B | EU269547 | BR69 |
| B78 | Get1A, Get1B | EU269548 | BR70 |
| B79 | MTA1A, MTA1B, MTA2B, MTA3A, MTA3B, MTA4A, MTA4B, Gua2A, Gua2B, Gua13A, Gua13B | EU269549 | BR71 |
| B80 | MTA2A | EU269550 | BR72 |
| *eliminated due to the presence of a large deletion. | | | |
